# Acute Tuberculosis of the Larynx*Reported to the Louisville Clinical Society.

**Published:** 1897-05

**Authors:** S. G. Dabney

**Affiliations:** Professor of Physiology and Clinical Lecturer on Ophthalmology, Otology, and Laryngology in the Hospital College of Medicine, etc., Louisville, Ky.


### ACUTE TUBERCULOSIS OF THE LARYNX.(?)*

BY S. G. DABNEY, M. D.

Professor of Physiology and Clinical Lecturer on Ophthalmology, Otology, and Laryngology in the Hospital College of Medicine, etc., Louisville, Ky.

The following case is of some interest, and I am sorry that I am not able to make a positive diagnosis. It is merely because of some obscure features that I report the case.

I was asked by a physician in the eastern part of the city to see with him a patient who was suffering with a sore throat. I found a lady fifty years of age who had a temperature of 103° to 105° F.; she complained of a great deal of pain at the

*Reported to the Louisville Clinical Society.

angle of the jaw on the right side and also just below the jaw. She had been sick but a few days, complaining of a sore throat and some fever, without any other definable symptoms. Her husband had died of typhoid fever a few weeks before but she presented no symptoms of the disease herself.

An examination of the throat showed a rather ugly looking ulcer around the third molar tooth of the right side; there was a little tendency to sloughing, but nothing especially characteristic. What evidently gave her the most trouble was an ulcer on the lingual surface of the epiglottis about as large as the average pea, somewhat irregular in outline, not deep, not undermined, rather yellowish in appearance and very painful to the touch. There were one or two other ulcers besides this large one, situated on the epiglottis.

I advised the application of a solution of cocaine to ease the pain on swallowing, and nitrate of silver applied once a day with mop and a local cleansing spray. I saw the patient again in two days; fever continued 103° to 104° and sometimes 105° F. At the second examination swelling had so increased that it was difficult to get a view of the interior of the larynx with the laryngoscope. A close examination showed that the arytenoid cartilages were dotted with a large number of minute ulcers not much larger than the head of a pin. I advised the physician in attendance to have a microscopic examination made of the sputum, thinking possibly it was a case of acute tuberculosis, which is still my judgment, although it certainly ran an unusual course for acute tuberculous infection of the larynx or any other part of the body. I was summoned to Virginia a few days later and did not have an opportunity of seeing the case again. She died four days afterwards. I understand that examination of the sputum was negative so far as finding the tubercle bacillus is concerned, a sample of the secretion having been obtained from the surface of the larynx by taking a swab of absorbent cotton and collecting a small quantity of the secretion in this way. In my judgment it is a rare case of acute tuberculous infection, notwithstanding the microscopist's report, though I am not positive.

Dr. T. P. SATTERWHITE: "The fact that the report of the microscopist was negative reminds me of a case that I saw on the fourth day of July last. The patient was a young girl, very robust looking, who had some months before passed through an attack of typhoid fever. She went off to the Springs to recuperate, and came back in first-class condition so far as I could see. A short time after her return she began having chilly sensations, elevation of temperature, rapid pulse, no rales or dullness over any part of the chest when I saw her, but in the course of several weeks rales made their appearance, and dullness was evident over the upper part of one lung, involving the entire lung in the course of three or four weeks from the time it first commenced. Her sputum was examined on four or five occasions by a competent microscopist of this city who pronounced the case not tuberculous, in any event he failed to find any tubercle bacilli. I diagnosed the case as one of acute tuberculosis, and requested the family to send for another physician. The next doctor who was called took some of the sputum and had no difficulty in finding an abundance of tubercle bacilli, although the other microscopist said he was unable to find any. It is a little singular that the first microscopist, who is certainly competent, was unable to discover any tubercle bacilli, when the other doctor, a general practitioner, whom, I presume, is competent also, had no difficulty in discovering an abundance of bacilli. I think it is well where you suspect tuberculosis to collect specimens of the sputum at various times and submit to different microscopists for examination."

Dr. LOUIS FRANK: "Speaking of the subject from the standpoint of bacteriology: The negative diagnosis in a case of this kind really does not mean as much as is generally supposed. It does not mean necessarily that the patient is not suffering from tuberculosis. The tubercle bacilli for certain reasons may be so diminished in quantity that none can be found in the slides examined; again, the sample of sputum may be so mixed with saliva, etc., the quantity placed under the microscope being very small, the portion actually examined might not contain the bacilli; on the other hand we might overlook organisms which were really present. In suspected cases of tuberculosis a number of slides should be examined. Even forty or fifty preparations have been examined without a single bacillus having been discovered, and perhaps in the next one we might find an abundance. We should examine the sputum repeatedly, as Dr. Satterwhite suggests, and while we may not find the bacilli at one time we might at another. For instance, in certain forms of kidney diseases, an abnormality of the urine may exist at one time and be absent at another, so that it would be necessary to make frequent and repeated examinations to make a diagnosis. The same statement applies with equal force in examining the sputum for the tubercle bacillus.

"In the case reported by Dr. Dabney it would be particularly difficult to discover the bacillus in an examination of the sputum, because if there was much secretion, the sputum being mixed with this secretion and the saliva expectorated, the organisms present in a small sample would be comparatively few, and repeated examinations might be necessary before bacilli would be discovered. In such a case the best plan would be to obtain a sample of the secretion by taking a small probe and wrapping it with absorbent cotton, pass it down into the ulcer and mop off some of the secretion from the surface of the ulcer, making a cover glass preparation of the sample thus obtained. That the tubercle bacillus is not found does not necessarily mean that the patient has not tuberculosis. Of course the positive finding is of the utmost value and settles the matter for all time. The repeated negative finding, however, I should say in examining the sputum, would be of considerable value in making a diagnosis."

